# Efficacy and safety of traditional Chinese medicine as an adjuvant to postoperative chemotherapy in colorectal cancer: a meta-analysis

**DOI:** 10.3389/fonc.2025.1700525

**Published:** 2026-01-22

**Authors:** Qinsi He, Xiaodan Chen, Haotian Zeng, Xinyu Gao, Zhi Zheng, Jun Rao, Qun Wen, Xuchao Yu, Jiquan Zeng

**Affiliations:** 1 Department of Integrated Traditional Chinese and Western Medicine, Jiangxi Cancer Hospital & Institute (The Second Affiliated Hospital of Nanchang Medical College), Nanchang, Jiangxi, China; 2 Department of Anorectal Surgery, Affiliated Hospital of Jiangxi University of Traditional Chinese Medicine, Nanchang, Jiangxi, China; 3 Department of Integrated Traditional Chinese and Western Medicine, Jiangxi Provincial People’s Hospital, Nanchang, Jiangxi, China

**Keywords:** adjuvant chemotherapy, colorectal cancer, meta-analysis, TCM, traditional Chinese medicine

## Abstract

**Objective:**

To systematically evaluate the efficacy and safety of traditional Chinese medicine (TCM) for postoperative adjuvant chemotherapy for colorectal cancer.

**Methods:**

CNKI, VIP, Wanfang, CBM, PubMed, and Web of Science were searched for the randomized controlled trials (RCT) of TCM participating in postoperative adjuvant chemotherapy for colorectal cancer. The search period was from January 1, 2018 to December 31, 2024. Cochrane bias risk assessment tool was used to evaluate the quality of included studies, and RevMan5.4 was used for meta-analysis.

**Results:**

A total of 41 randomized controlled trials involving 2918 patients with colorectal cancer was ultimately included. The results demonstrated that the combination of TCM with chemotherapy was superior to chemotherapy alone in several aspects. These included the objective response rate (ORR), improvement of TCM-related symptoms, levels of tumor markers CEA and CA199, immune function indicators (CD3^+^, CD4^+^, CD4^+^/CD8^+^, NK cells), and quality of life as measured by the KPS score. Additionally, the combination therapy reduced CD8^+^ levels and mitigated abnormal laboratory indicators caused by chemotherapy, such as leukopenia, thrombocytopenia, decreased hemoglobin, and abnormal liver and kidney function. Furthermore, it alleviated chemotherapy-related adverse effects (AEs), including nausea, vomiting, and peripheral nerve toxicity.

**Conclusions:**

TCM may be associated with improvements in quality of life and reduce chemotherapy side effects in postoperative colorectal cancer patients, though large-scale rigorous trials are needed to confirm efficacy and safety.

**Systematic Review Registration:**

https://www.crd.york.ac.uk/prospero/, identifier CRD42025635900.

## 1 Introduction

Colorectal cancer (CRC) is one of the most commonly diagnosed malignancies worldwide and remains the third leading cause of cancer-related mortality. According to recent global cancer statistics, the incidence and mortality of CRC have shown a steady upward trend, particularly in low- and middle-income countries where urbanization, aging populations, and lifestyle shifts contribute to an increasing disease burden (1). The development of CRC is influenced by both modifiable and non-modifiable risk factors. Approximately 70–75% of cases are sporadic and linked to modifiable factors such as unhealthy diets, physical inactivity, obesity, smoking, and alcohol consumption. The remaining 25–30% arise from non-modifiable factors, including advanced age, family history, a personal history of colorectal adenomas or inflammatory bowel disease, and hereditary syndromes such as Lynch syndrome or familial adenomatous polyposis (1). Despite significant advancements in diagnostic and therapeutic approaches, prognosis remains stage-dependent and highly variable across regions. In high-income countries, the 5-year relative survival rate exceeds 65%, while in low-resource settings, it remains below 50% (2, 3).

Surgical resection followed by postoperative adjuvant chemotherapy is the standard treatment for early- and intermediate-stage CRC. However, recurrence rates remain concerning, affecting approximately 16% of stage II and up to 40% of stage III patients (4). This is often attributed to the inability of chemotherapy to eliminate microscopic residual disease and the adverse effects that lead to poor treatment compliance. Therefore, optimizing current treatment strategies through adjunctive approaches is of critical importance.

Traditional Chinese Medicine (TCM), with its holistic and multi-targeted therapeutic philosophy, has been increasingly recognized as a valuable adjunct in cancer care. Preclinical studies have shown that TCM exerts anti-cancer effects via multiple mechanisms, including apoptosis induction, immune modulation, anti-inflammatory activity, oxidative stress reduction, and regulation of gut microbiota (5).

Clinically, TCM has demonstrated potential in improving cancer-related symptoms, enhancing patients’ quality of life, mitigating chemotherapy-induced toxicities (e.g., nausea, vomiting, hematologic suppression, and peripheral neuropathy), and supporting immune and organ function. Several studies also report improvements in traditional Chinese medicine syndrome scores, Karnofsky Performance Status (KPS), tumor marker levels (CEA, CA19-9), and immune indices (CD3+, CD4+, CD8+, NK cells) following TCM-assisted chemotherapy (6, 7).

Nevertheless, existing clinical evidence remains fragmented, and a comprehensive synthesis of high-quality data is lacking. Therefore, this study aims to systematically evaluate the efficacy and safety of TCM as an adjuvant to postoperative chemotherapy in non-advanced colorectal cancer through a meta-analysis. By quantitatively analyzing objective response rates, symptom scores, immune and laboratory parameters, and chemotherapy-related toxicities, this study seeks to provide robust evidence to support the integration of TCM into standardized colorectal cancer management.

## 2 Materials and methods

This systematic review and meta-analysis were conducted following the PRISMA (Preferred Reporting Items for Systematic Reviews and Meta-Analyses) guidelines to ensure methodological rigor and transparency (8). The study protocol was prospectively registered with the International Prospective Register of Systematic Reviews (PROSPERO), under the registration number CRD42025635900.

### 2.1 Eligibility criteria

The inclusion and exclusion criteria of this meta-analysis were formulated based on the PICOS framework. Eligible studies were randomized controlled trials (RCTs) involving patients with pathologically confirmed colorectal cancer who underwent postoperative adjuvant chemotherapy, regardless of gender, race, or geographic region. In the intervention group, patients received TCM in combination with standard postoperative adjuvant chemotherapy, with no restriction on the type or formulation of TCM. The control group received postoperative adjuvant chemotherapy alone. Studies were excluded if they involved patients with unclear tumor staging, CRC with metastasis, absence of adjuvant chemotherapy, non-primary colorectal cancer or concurrent malignancies, or if they were non-RCTs, lacked key outcome data, or did not provide sufficient statistical information for inclusion.

### 2.2 Outcomes

The primary outcomes of this study encompassed both efficacy and safety indicators. Efficacy outcomes included objective response rate (ORR), quality of life (QoL), tumor marker levels, and immune function parameters. ORR was defined as the proportion of patients achieving complete response (CR) or partial response (PR), based on the World Health Organization (WHO) criteria or the Response Evaluation Criteria in Solid Tumors (RECIST); rates of stable disease (SD) and progressive disease (PD) were also reported when available. QoL was assessed using the Karnofsky Performance Status (KPS) score. Tumor markers evaluated included carcinoembryonic antigen (CEA) and carbohydrate antigen 19-9 (CA19-9). Immune function was measured by levels of CD3^+^, CD4^+^, and CD8^+^ T lymphocytes, the CD4^+^/CD8^+^ ratio, and natural killer (NK) cell counts. Safety outcomes comprised hematologic toxicity (anemia, thrombocytopenia, leukopenia), hepatic and renal dysfunction, gastrointestinal adverse events (nausea and vomiting), and peripheral neurotoxicity.

### 2.3 Search strategy and study selection

A comprehensive literature search was performed to identify relevant RCTs evaluating TCM combined with postoperative adjuvant chemotherapy for colorectal cancer. The search was conducted independently by two reviewers (Qinsi He and Xinyu Gao) using both Medical Subject Headings (MeSH) terms and free-text keywords. The following electronic databases were searched from January 1, 2018, to December 31, 2024, Chinese Biomedical Literature Database (CBM), China National Knowledge Infrastructure (CNKI), Chongqing VIP Database (VIP), and Wanfang Data for Chinese databases, and PubMed and Web of Science for international databases.

The search strategy combined terms related to colorectal cancer (e.g., “colorectal cancer, “ “colorectal neoplasm, “ “colorectal tumors”), Traditional Chinese Medicine (e.g., “herbal medicine, “ “Chinese herbal medicine, “ “traditional Chinese medicine”), and study design (e.g., “randomized, “ “randomized controlled trial”). The detailed search strategies were shown in **Supplementary Table S1**. Boolean operators (AND, OR) were used to combine terms appropriately to maximize search sensitivity. The titles and abstracts of all retrieved records were screened for relevance, and full texts of potentially eligible articles were then assessed against the inclusion criteria. Any disagreements between the two reviewers were resolved by discussion with a third researcher (Xiaodan Chen) to reach consensus.

The search strategy was adapted to the specific requirements of each database to ensure comprehensive coverage. Reference lists of included studies and relevant reviews were also manually searched to identify additional eligible studies.

### 2.4 Data extraction

Data extraction was independently performed by two reviewers, and extracted information included the first author, year of publication, sample size, types of medications, cancer staging system (TNM stage), Karnofsky Performance Status (KPS), details of the TCM intervention, standard treatment regimen, and outcome measures. Any discrepancies were resolved through discussion and consensus.

### 2.5 Assessment of risk of bias

Quality assessment was performed according to the Cochrane Handbook criteria, categorizing risk of bias as low risk, some concerns, or unclear risk based on the evaluation of six domains: selection bias, performance bias, detection bias, attrition bias, reporting bias, and other potential biases. Two researchers independently conducted and cross-checked the quality assessment, with any disagreements resolved through discussion involving a third researcher.

### 2.6 Statistical analyses

Data were analyzed using RevMan 5.4 software. Categorical variables were presented as odds ratios (ORs) with 95% confidence intervals (CI), while continuous variables were expressed as mean differences (MDs) with 95% CI. Heterogeneity among studies was assessed using the I² statistic. When P > 0.1 and I² < 50%, heterogeneity was considered low, and a fixed-effects model was applied; otherwise, a random-effects model was used. Sensitivity analysis was conducted by excluding articles one by one. In addition, funnel plot, Begg test, and Egger test were used to detect publication bias.

## 3 Results

### 3.1 Literature search and basic characteristics

**Figure 1:**
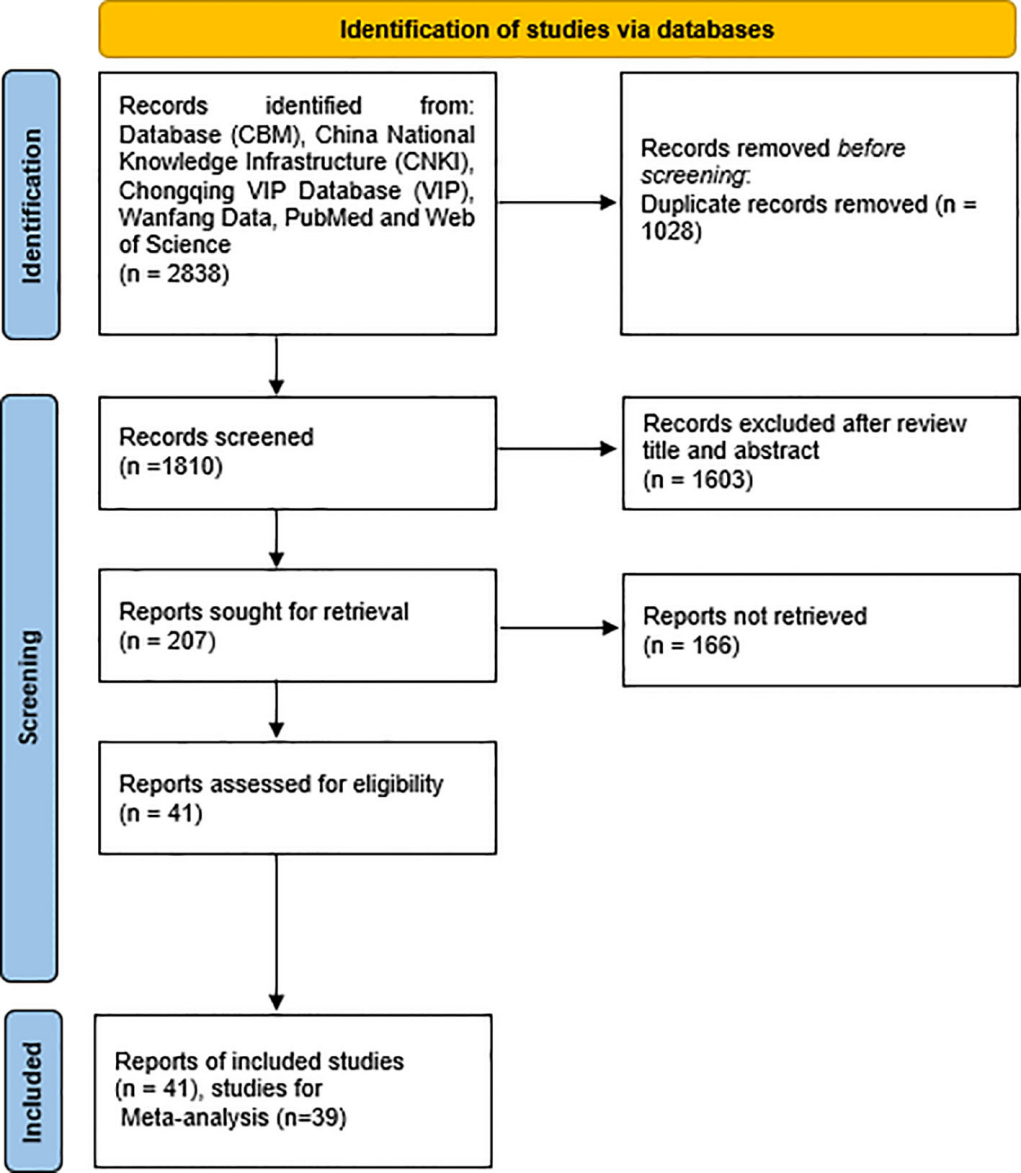
The flow chart of included studies.

A total of 2, 838 records were initially retrieved from six major, after removing 1, 028 duplicates, 1, 810 records remained for title and abstract screening. Ultimately, 41 RCTs met eligibility criteria and were included in the final meta-analysis (**Figure 1**). A total of 41 RCTs published between 2018 and 2024 were included in this study, involving postoperative patients with colorectal cancer. The experimental groups received oral TCM interventions—such as formulas for tonifying qi and spleen, resolving phlegm, or detoxifying the kidney—while the control groups were treated with standardized chemotherapy regimens, including FOLFOX, XELOX, or CapeOX. Treatment durations generally ranged from 2 to 12 cycles. Most participants were diagnosed with stage II–III CRC, with some studies also including stage I or high-risk stage II cases. Reported outcomes encompassed tumor efficacy, tumor markers (e.g., CEA, CA199), improvement in TCM syndromes, immune function, quality of life (measured by KPS score), laboratory parameters, and chemotherapy-related AEs, reflecting the multidimensional clinical effects of integrative therapy. The detailed information was shown in **Table 1**.

**Table 1:** Characteristics of the included studies.

stud	year	Sample size	Gender		Therapeutic measures			Tumor stage/kps		Treatment time	Outcomes
			Experimental group (T)	Control group (C)	Experimental group	Chinese medicine prescription	Control group	Tumor stage (non-advanced (stage I-III)	Group		
Zeng di	2023	40/40	25/15	23/17	Phlegm-Resolving and Mass-Dispersing Formula, po, 150ml/time, bid, 3 weeks/cycle, 6 cycles	Fabanxia and tiannanxing each 15g, quanxie, fuling, wugong, huangqi, jineijin, shancigu each 12g, shanyao,zhishi, yiyiren each 10g, zhigancao 8g	Xelox chemotherapy regimen: OX130mg/m2, ivgtt, 2h, d1; capecitabine 1250mg/m2, bid, po, d1-d14,3 weeks/cycle, 6 cycles	CRC: II (28/30); III (12/10)	A	Oral, 21d,6 cycles	2,3,4,6,7
Chen Chaoyang	2021	30/30	16/14	December 18	Yiqi Jianpi Decoction, po, 150ml/ time, bid, take for 14 days, stop for 7 days as a course of treatment, 2 courses	Renshen, dangshen,baishi , and Hanfang each 10g, huangqi and gancao each 30g, shaoyao 60g	FOLFOX6 chemotherapy regimen: OX 85 mg/m², ivgtt, d1; CF200 mg/m², ivgtt, d1-d2; fluorouracil 500 mg/m², intravenous injection, d1; followed by 2500 mg/m², ivgtt, 48 h. 3 weeks/cycle, 2 cycles.	CRC: IIA (2/1), IIB (6/5), IIIA(5/4), IIIB (17/20); kps>50	A	Oral, 14 days (21 days), 2 cycles	1,2,3

### 3.2 Risk of bias assessment for included studies

Characteristics and quality of all included studies are presented in **Supplementary Figures 1, 2**.

### 3.3 Tumor objective efficacy - objective response rate

**Figure 2:**
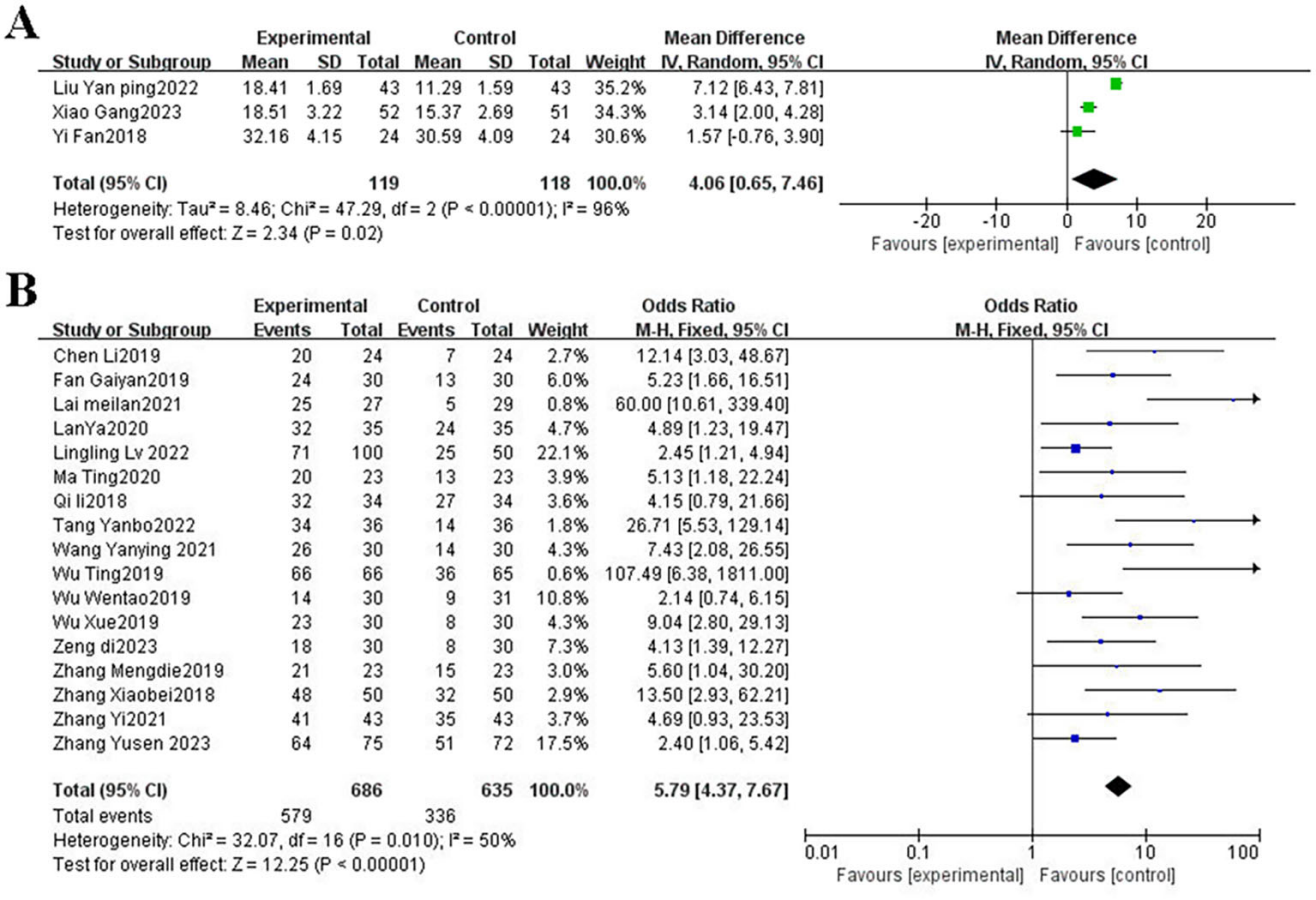
**(A)** The forest plot of objective response rate; **(B)** Forest plot of the total effective rate of TCM syndrome improvement. TCM, traditional Chinese medicine.

The efficacy evaluation followed the WHO or RECIST 1.1 criteria, categorizing outcomes into complete response (CR), partial response (PR), stable disease (SD), and progressive disease (PD). The ORR was calculated as: ORR=(CR+PR) cases/Total Cases×100%. A total of three RCTs (9–11), including 263 patients, reported on objective response rate. Heterogeneity among the studies was not statistically significant (*P* = 0.52, I² = 0.000%), indicating consistency across trials; therefore, a fixed-effects model was applied for the pooled analysis. The meta-analysis demonstrated that the combination of TCM with postoperative adjuvant chemotherapy significantly improved ORR compared with chemotherapy alone (OR = 3.32, 95% CI: 1.99–5.33, Z = 4.59, *P* < 0.001). These results suggested that TCM as an adjunct to postoperative adjuvant chemotherapy may improve the therapeutic response in patients with CRC (**Figure 2A**).

### 3.4 The total effective rate of TCM syndrome improvement

TCM symptom scoring criteria referred to the Guiding Principles for Clinical Research of New Chinese Medicine, which were divided into obvious, effective, ineffective, total effective rate (%) = (obvious + effective) cases/total cases ×100%. 17 RCTs (12–28) mentioned the total effective rate of TCM syndrome improvement. The pooled results demonstrated a significant difference between the two groups (OR = 5.79, 95% CI: 4.37–7.67, *P* < 0.001; I² = 50%; **Figure 2B**). Compared with postoperative adjuvant chemotherapy alone, the combination of TCM and postoperative adjuvant chemotherapy was associated with a markedly greater improvement in TCM syndrome scores among patients with colorectal cancer.

### 3.5 Tumor marker levels—CEA and CA19-9

**Figure 3:**
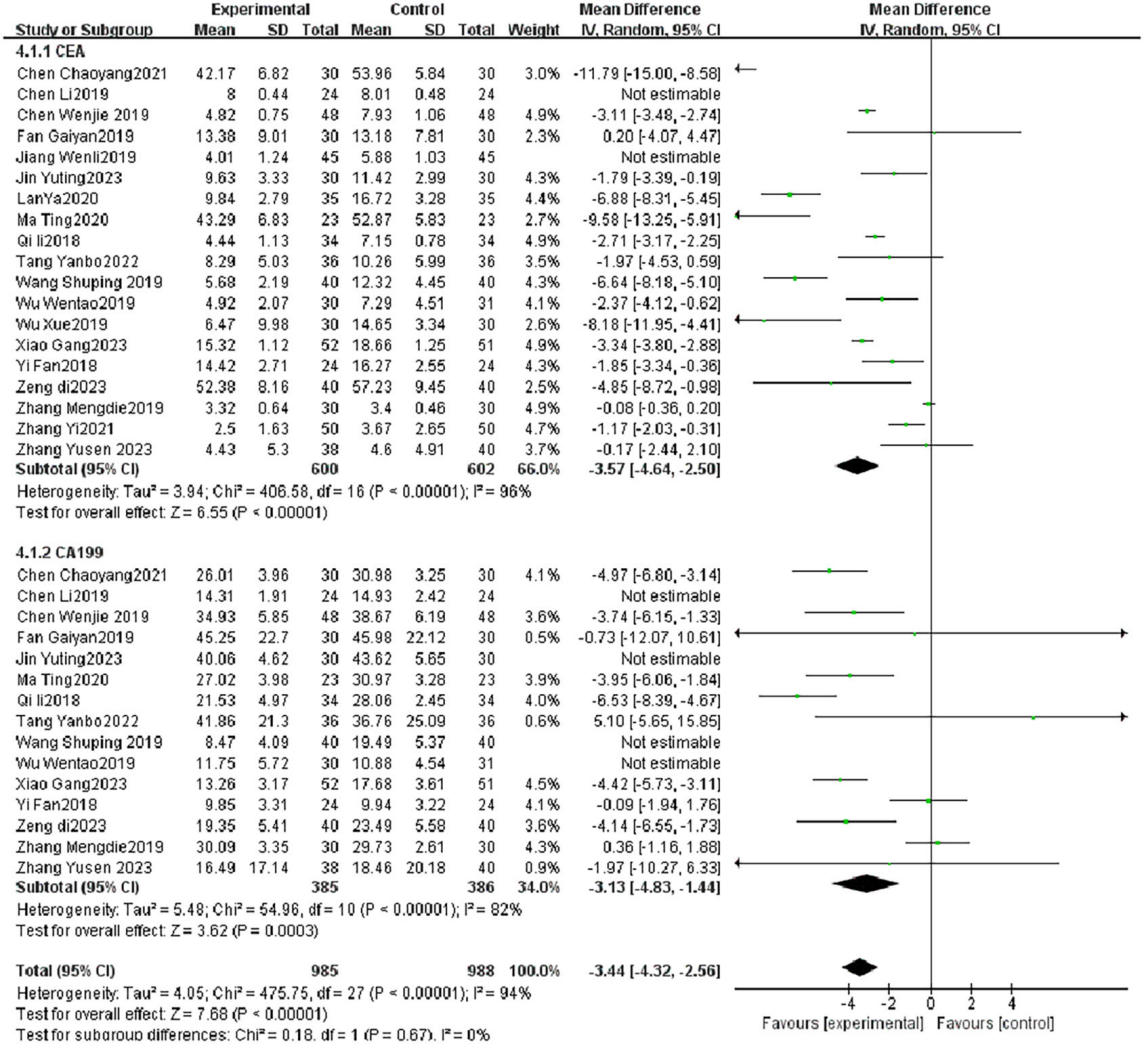
The forest plot of tumor marker levels (CEA and CA19-9) comparing TCM plus chemotherapy versus chemotherapy alone. Data presented as mean difference (MD) with 95% confidence intervals. Negative values favor the intervention group. CEA, carcinoembryonic antigen; CA19-9, carbohydrate antigen 19-9; TCM, traditional Chinese medicine.

After combining 19 studies (10–13, 15, 17–19, 22–25, 27–33)of CEA and 15 studies (10–13, 17, 19, 22, 24, 25, 28–33) of CA199 (**Figure 3**), TCM combined with postoperative adjuvant chemotherapy group exerted a better protective effect on tumor marker lever (CEA MD = -3.57, 95% CI = -4.64 to -2.5, *P* < 0.001, CA199 MD = -3.13, 95% CI = -4.83 to -1.44, *P* < 0.001) than control group. However, CEA I^2^ = 96% and CA199 I^2^ = 82%, representing large heterogeneity, Sensitivity analysis was performed for each outcome, and no significant source of heterogeneity was found during the analysis, and the results of meta-analysis showed good stability and statistical significance (*P* < 0.001). This indicated that TCM combined with postoperative adjuvant chemotherapy group significantly reduced the tumor markers lever (CEA, CA199) compared with the control group.

### 3.6 Immune function

**Figure 4:**
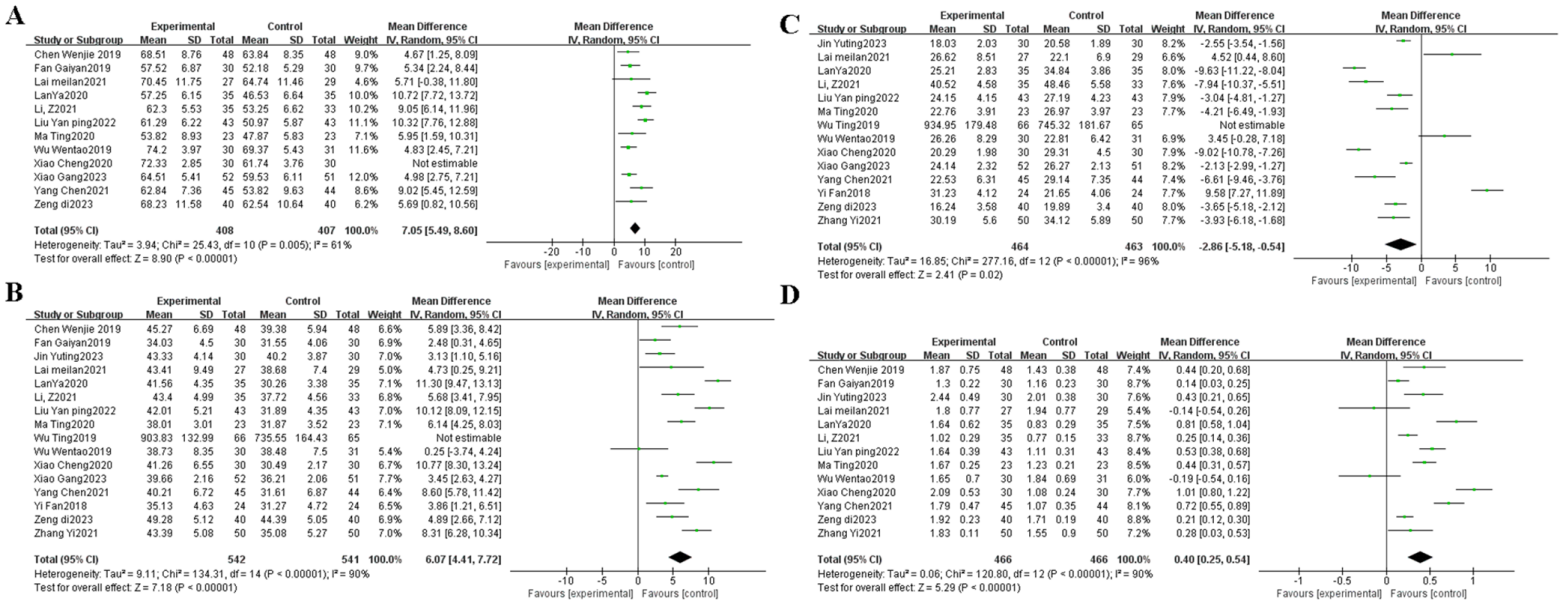
**(A)** The forest plot of CD3^+^; **(B)** The forest plot of CD4^+^; **(C)** The forest plot of CD8^+^; **(D)** The forest plot of the ratio of CD4^+^/CD8^+^.

There were 12, 16, 14, 13 and three RCTs contributed to the analysis of CD3^+^ (9, 10, 13–15, 17, 22, 24, 30, 34–36), CD4^+^ (9–11, 13–15, 17, 21, 22, 24, 27, 30, 32, 34–36), CD8^+^ (9–11, 14, 15, 17, 21, 22, 24, 27, 32, 34–36), CD4^+^/CD8^+^ (9, 13–15, 17, 22, 24, 27, 30, 32, 34–36), NK (9–11) respectively, representing statistical heterogeneity in CD3^+^ T cells (I^2^ = 61%), CD4^+^ T cells (I^2^ = 90%), CD8^+^ T cells (I^2^ = 96%), CD4^+^/CD8^+^ T cells ratio (I^2^ = 90%), and NK cells (I^2^ = 96%), hence, the random-effects model was adopted, The results of I² value and combined effect size showed no significant changes after removing literatures one by one, which proved that the study results were stable and reliable. The meta-analysis results showed that TCM plus postoperative adjuvant chemotherapy improved the CD3^+^ (MD, 7.05 [5.49, 8.60], *P* < 0.001) (**Figure 4A**), CD4^+^ (MD, 6.07 [4.41, 7.72], *P* < 0.001) (**Figure 4B**), CD4^+^/CD8^+^T cells ratio (MD, 0.40 [0.25, 0.54], *P* < 0.001) (**Figure 4C**), and NK cells (MD, 4.06 [0.65, 7.46], *P* = 0.002, **Supplementary Figure 3A**) and reduced the CD8^+^ (MD, -2.86 [-5.18, -0.54], *P* = 0.020) (**Figure 4D**) compared with postoperative adjuvant chemotherapy alone.

### 3.7 Quality of life KPS score

The quality of life was evaluated according to the Karnofsky (KPS) patient health status rating scale, and evaluated once before and after treatment. The heterogeneity analysis of the result contained a total of 16 studies (10, 12, 18, 19, 21–23, 28, 31, 34, 35, 37–40, 44) included 1209 patients showed I^2^ = 95%, and the random-effect model was applied, the meta-analysis showed good stability and statistical significance (*P* < 0.001). Compared with the control group treated with postoperative adjuvant chemotherapy alone, quality of life KPS score of non-advanced colorectal cancer patients in the treatment group treated with TCM combined with postoperative adjuvant chemotherapy was significantly improved. [MD = 6.75, 95%CI (4.65, 8.86), *P* < 0.001] (**Supplementary Figure 3B**).

### 3.8 Laboratory examination

There were 12, 11, 15, and 4 RCTs contributed to the analysis of leukopenia (9, 13, 15, 19, 23, 28, 30, 36, 41–44), hemoglobin (13, 19, 23, 24, 28, 41–46), thrombocytopenia (9, 13, 15, 23, 24, 27, 28, 30, 36, 41–46), abnormal liver and kidney function respectively (15, 30, 40, 42), representing low heterogeneity in leukopenia (I^2^ = 24%), hemoglobin (I^2^ = 26%), thrombocytopenia (I^2^ = 0), and abnormal liver and kidney function (I^2^ = 0), hence, the fixed-effects model was adopted. The meta-analysis results showed that TCM plus postoperative adjuvant chemotherapy reduced the risk of the leukopenia (OR, 0.39 [0.29, 0.53], *P* < 0.001), hemoglobin (OR, 0.50 [0.37, 0.67], *P* < 0.001), thrombocytopenia (OR, 0.49 [0.36, 0.65], *P* < 0.001) and abnormal liver and kidney function (OR, 0.31 [0.14, 0.67], *P* = 0.003) compared with postoperative adjuvant chemotherapy alone (**Supplementary Figures 4A–D**).

### 3.9 Adverse reactions

#### 3.9.1 Nausea and vomiting

16 RCTs (11–13, 15, 17, 19, 23, 24, 27, 28, 30, 34, 40, 43, 45, 47) mentioned AEs nausea and vomiting, owing to the low heterogeneity, the fixed effect model was selected for further calculation, and the results showed significant difference between two groups (OR, 0.34 [0.25, 0.47], *P* < 0.001, I^2^ = 30%). Compared with postoperative adjuvant chemotherapy group, TCM combined with postoperative adjuvant chemotherapy group could reduce the incidence of AEs nausea and vomiting of CRC patients (**Supplementary Figure 5**).

#### 3.9.2 Peripheral nerve toxicity

13 RCTs (12, 15, 17, 19, 22, 23, 28, 31, 37, 41, 43–45) mentioned AEs peripheral nerve toxicity, owing to the low heterogeneity, the fixed effect model was applied, showed significant difference between two groups (OR, 0.49 [0.35, 0.67], *P* < 0.001, I^2^ = 26%). Indicating that in the treatment of CRC, compared with postoperative adjuvant chemotherapy group, TCM combined with postoperative adjuvant chemotherapy group could reduce the incidence of AEs peripheral nerve toxicity (**Supplementary Figure 6**).

## 4 Discussion

This meta-analysis of 41 trials involving 2918 CRC patients without metastasis demonstrates that the integration of TCM with postoperative adjuvant chemotherapy offers significant clinical advantages over chemotherapy alone. TCM significantly enhanced tumor response, improved immune function, reduced tumor marker levels (CEA, CA19-9), and mitigated treatment-related toxicities. Notably, it also contributed to better quality of life and a lower incidence of hematologic and organ toxicities, highlighting its potential as a safe and effective adjunct to conventional therapy.

Despite advances in surgical techniques and adjuvant therapies, CRC recurrence remains a major clinical challenge. Recurrence is primarily driven by minimal residual disease, adverse tumor biology (e.g., RAS/BRAF mutations, MSS status), lymphovascular invasion, inadequate resection margins, and non-adherence to postoperative treatment or follow-up (48, 49). Strategies to reduce recurrence include high-quality standardized surgery, risk-adapted adjuvant therapy, and rigorous surveillance. Although surgical resection, chemotherapy, radiotherapy, and targeted therapies have significantly improved patient survival, recurrence remains a critical factor affecting long-term prognosis. Increasing evidence suggests that integrating TCM with postoperative adjuvant chemotherapy may offer clinical benefits in CRC management. TCM has shown potential in alleviating chemotherapy-induced toxicity, improving gastrointestinal symptoms, enhancing immune function, and modulating tumor microenvironment (50, 51).

Our results showed that TCM involvement in adjuvant chemotherapy was positive and aligned with the findings of the following studies on TCM in cancer (50, 52). Researches had consistently shown that TCM not only relieved symptoms like fatigue, chronic pain, anorexia/cachexia, and insomnia in cancer patients, enhancing their QOL, but also mitigated the side effects and complications caused by chemotherapy, radiotherapy, and targeted treatments (53). Moreover, TCM possessed anti-tumor properties, Sijunzi Decoction could promote apoptosis and autophagy of CRC cells through PI3K/Akt/mTOR pathway (54). However, Other systematic studies focus on the combination of TCM with specific chemotherapy drugs, the use of TCM in injectable forms, and its efficacy and safety in managing specific chemotherapy-induced side effects, such as diarrhea (50, 55, 56). The outcome indicators in our study were consistent with those reported in previous research. Tumor response, reflected by improvements in ORR, was enhanced with the addition of TCM. Common AEs associated with postoperative adjuvant chemotherapy—such as nausea, vomiting, and peripheral neurotoxicity—were significantly reduced. Hematologic and organ function safety profiles also improved, with lower incidences of leukopenia, anemia, thrombocytopenia, and abnormal liver and kidney function. Furthermore, immune parameters, including elevated CD3^+^, CD4^+^, and CD4^+^/CD8^+^ ratios, alongside reduced CD8^+^ levels, indicated a shift toward an activated, non-immunosuppressive state (57). This immune modulation may enhance immune surveillance and reduce the risk of tumor recurrence and metastasis (58). The interpretation of these findings requires careful clinical context. While CD8^+^ cytotoxic T cells are crucial for anti-tumor immunity, their functional state—rather than their absolute number alone—determines their overall effect. A depressed immune state, often induced by chemotherapy, is characterized not only by low CD4^+^ counts but also by an inverted CD4^+^/CD8^+^ ratio. Therefore, the key finding is the restoration of a more balanced CD4^+^/CD8^+^ ratio, which is a recognized indicator of improved overall immune competence and is often associated with better clinical outcomes in cancer patients.

sCrucially, these favorable shifts in immune parameters were observed alongside direct patient benefits documented in our analysis: namely, improved control of chemotherapy-related symptoms (e.g., nausea, vomiting), enhanced quality of life (KPS score), and a reduction in hematologic toxicity. This correlation suggests that the immunomodulatory effects of TCM are part of a broader biological response that may contribute to better treatment tolerance and patient well-being. It should be noted that the decrease in CD8^+^ T cells could reflect a reduction in exhausted or regulatory subsets; however, in the absence of more specific T-cell phenotyping data, we refrain from concluding a direct anti-tumor benefit from the CD8^+^ decrease alone. The collective improvement in multiple immune subsets and, more importantly, their correlation with tangible clinical endpoints, supports the potential role of TCM in mitigating chemotherapy-induced immunosuppression.

Despite providing supportive evidence for the efficacy and safety of TCM as an adjuvant to postoperative chemotherapy in CRC, this study has several limitations. First, substantial heterogeneity was observed across the included trials, arising from differences in TCM formulations, individual herbal components, chemotherapy regimens, treatment durations, follow-up periods, and definitions of clinical outcomes. This heterogeneity may reduce the reliability and consistency of the pooled estimates. Additionally, potential publication and selection biases, particularly the underreporting of negative or inconclusive results in TCM research, may have skewed the findings. Second, many studies had small sample sizes and lacked rigorous methodological design, limiting the statistical power and external validity of the results. Furthermore, a key limitation of this study is the inability to perform a stratified analysis by tumor stage, as the included studies predominantly involved patients with stages I-III without providing sufficiently granular data for distinct stage-based comparisons.

Future studies should focus on the standardization of TCM interventions for specific clinical stages of CRC, including the selection of dosage forms (e.g., injections, decoctions, or granules), drug categories (e.g., tonifying or detoxifying agents), dosage, duration, and optimal initiation timing. Well-designed, large-scale, multicenter RCTs are needed to further confirm the clinical benefits and safety profile of TCM. Moreover, mechanistic studies should be strengthened to elucidate the pharmacological basis of TCM interventions and support evidence-based clinical application.

## 5 Conclusions

TCM may be a complementary approach that warrants further investigation for its potential to improve QOL and reduce chemotherapy-related AEs. Existing studies indicate that TCM interventions might contribute to QOL improvement, mitigation of chemotherapy-related AEs, and immunomodulatory benefits. However, it should be emphasized that current evidence remains insufficient to draw definitive conclusions, as most available studies are limited by small sample sizes, methodological heterogeneity, and potential publication bias. Large-scale, rigorously designed RCTs with standardized outcome measures are required to establish robust evidence regarding the efficacy, safety profiles, and underlying mechanisms of TCM in this clinical context.

## Data Availability

The original contributions presented in the study are included in the article/**Supplementary Material**. Further inquiries can be directed to the corresponding author.
