# Supplementary material for: Efficacy and safety of traditional Chinese medicine as an adjuvant to postoperative chemotherapy in colorectal cancer: a meta-analysis
Source: Front Oncol. 2026 Jan 22;15:1700525. doi: 10.3389/fonc.2025.1700525 (PMC12878152; doi:10.3389/fonc.2025.1700525)
Supplement: Supplementary file 1 [file DataSheet1.docx]

Supplementary Material

**Supplementary Figures**


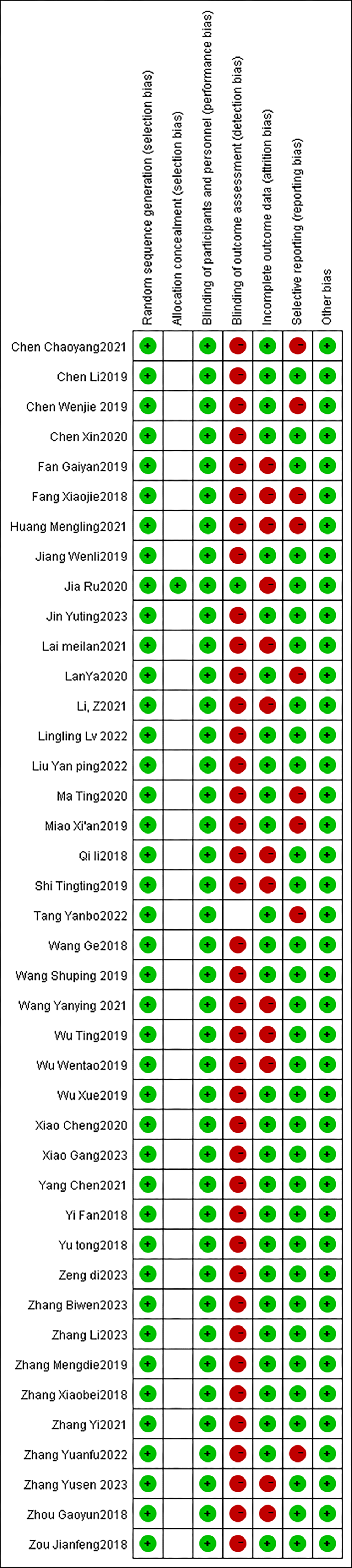


Supplementary Figure 1: The risk bias assessment graph.





Supplementary Figure 2: The risk bias assessment summary.


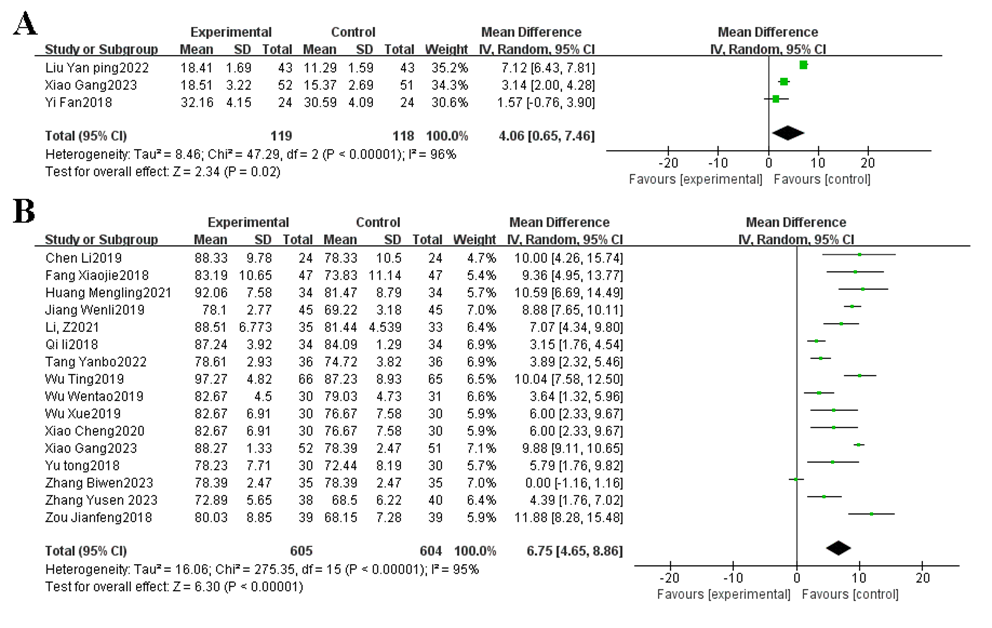


Supplementary Figure 3: 3A: The forest plot of the ratio of NK; 3B: The forest plot of KPS score.


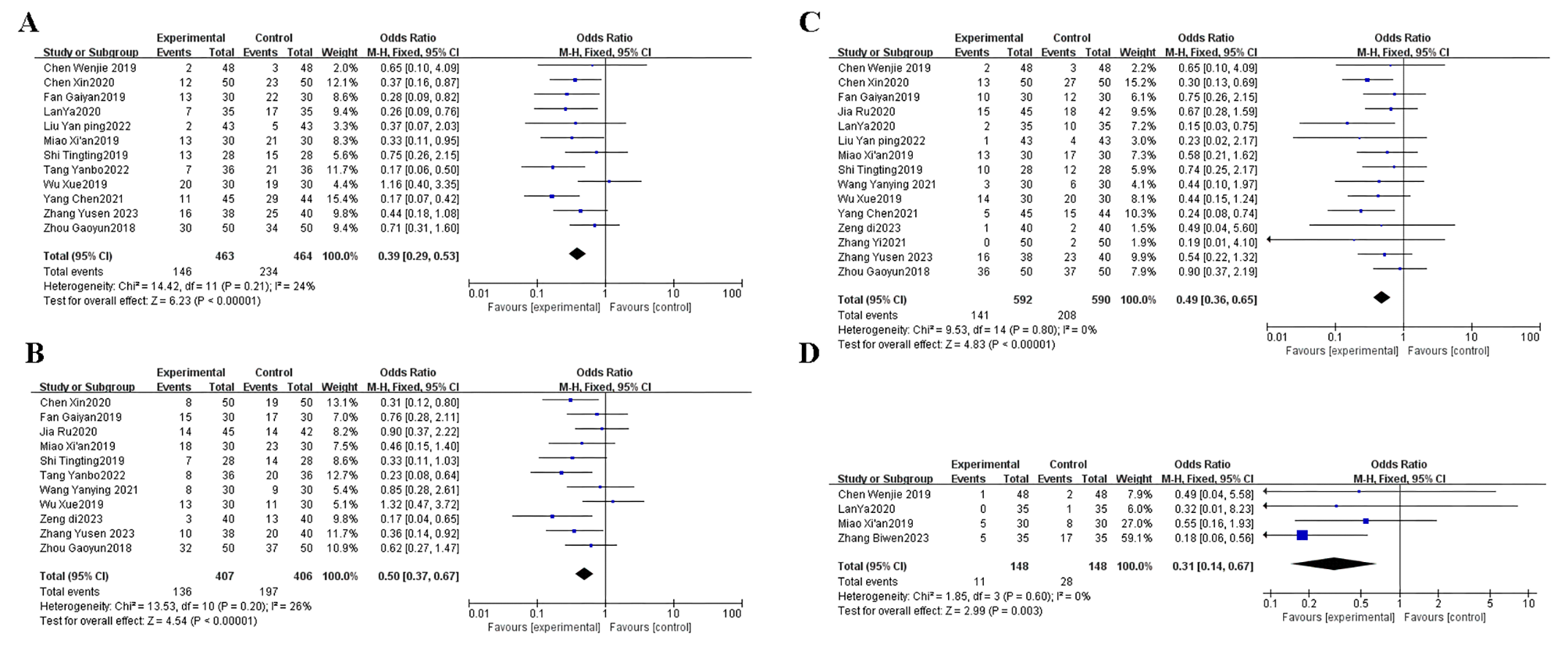


Supplementary Figure 4: 4A: The forest plot of Leukopenia; 4B: The forest plot of decreased hemoglobin; 4C: The forest plot of thrombocytopenia; 4D: The forest plot of abnormal liver and kidney function.





Supplementary Figure 5: The forest plot of nausea and vomiting.





Supplementary Figure 6: The Forest plot of peripheral nerve toxicity.
