# Supplementary material for: Efficacy and safety of traditional Chinese medicine as an adjuvant to postoperative chemotherapy in colorectal cancer: a meta-analysis
Source: Front Oncol. 2026 Jan 22;15:1700525. doi: 10.3389/fonc.2025.1700525 (PMC12878152; doi:10.3389/fonc.2025.1700525)
Supplement: Supplementary file 2 [file DataSheet2.docx]

| **Databases** | **Search strategy** |
| --- | --- |
| **CNKI/VIP/万方** | (主题=("结直肠癌" OR "结直肠肿瘤" OR "结肠癌" OR "直肠癌" OR "大肠癌") AND 主题=("术后化疗" OR "辅助化疗") AND 主题=("中医药" OR "中药" OR "中草药" OR "传统中医" OR "中西医结合" OR "汤剂" OR "注射液" OR "中成药")) AND 主题=("随机" OR "RCT") |
| **CBM** | ("结直肠癌"[常用字段:智能] OR "结肠癌"[常用字段:智能] OR "直肠癌"[常用字段:智能]) AND ("术后化疗"[常用字段:智能] OR "辅助化疗"[常用字段:智能]) AND ("中医药"[常用字段:智能] OR "中药"[常用字段:智能]) AND "随机对照试验"[分类] |
| **PubMed** | (("Colorectal Neoplasms"[Mesh] OR colorectal cancer[tiab] OR colorectal carcinoma[tiab] OR colon cancer[tiab] OR rectal cancer[tiab])) AND (("Drugs, Chinese Herbal"[Mesh] OR "Medicine, Chinese Traditional"[Mesh] OR Chinese herbal[tiab] OR Traditional Chinese Medicine[tiab] OR TCM[tiab])) AND (("Chemotherapy, Adjuvant"[Mesh] OR postoperative chemotherapy[tiab] OR adjuvant chemotherapy[tiab])) AND (randomized controlled trial[pt] OR randomized[tiab] OR randomised[tiab] OR RCT[tiab]) |
| **Web of Science** | TS= (("colorectal cancer" OR "colorectal neoplas" OR "colon cancer" OR "rectal cancer") AND ("Traditional Chinese Medicine" OR "Chinese herbal" OR "TCM" OR "herb") AND ("postoperative chemotherapy" OR "adjuvant chemotherapy") AND (random* OR RCT)) |

**The detailed search strategy of electronic databases.**

**Abbreviation：CBM:**

**Chinese Biomedical Literature Database, CNKI: China National Knowledge Infrastructure, VIP: Chongqing VIP Database (VIP)**
